# Emergence of multicluster chimera states

**DOI:** 10.1038/srep12988

**Published:** 2015-09-09

**Authors:** Nan Yao, Zi-Gang Huang, Celso Grebogi, Ying-Cheng Lai

**Affiliations:** 1 Department of Applied Physics, Xi’an University of Technology, Xi’an 710054, China; 2 School of Electrical, Computer and Energy Engineering, Arizona State University, Tempe, AZ 85287, USA; 3 School of Physical Science and Technology, Lanzhou University, Lanzhou 730000, China; 4 Institute for Complex Systems and Mathematical Biology, King’s College, University of Aberdeen, Aberdeen AB24 3UE, United Kingdom; 5 Department of Physics, Arizona State University, Tempe, Arizona 85287, USA

## Abstract

A remarkable phenomenon in spatiotemporal dynamical systems is chimera state, where the structurally and dynamically identical oscillators in a coupled networked system spontaneously break into two groups, one exhibiting coherent motion and another incoherent. This phenomenon was typically studied in the setting of non-local coupling configurations. We ask what can happen to chimera states under systematic changes to the network structure when links are removed from the network in an orderly fashion but the local coupling topology remains invariant with respect to an index shift. We find the emergence of multicluster chimera states. Remarkably, as a parameter characterizing the amount of link removal is increased, chimera states of distinct numbers of clusters emerge and persist in different parameter regions. We develop a phenomenological theory, based on enhanced or reduced interactions among oscillators in different spatial groups, to explain why chimera states of certain numbers of clusters occur in certain parameter regions. The theoretical prediction agrees well with numerics.

The collective behaviors of systems of coupled oscillators have been a topic of continuous interest^1^,^2^,^3^. A class of oscillator systems is those with non-local interactions, which arise in realistic systems such as Josephson-junction arrays^4^ and chemical oscillators^5^,^6^. A phenomenon of recent interest is chimera states^7^,^8^,^9^,^10^,^11^,^12^,^13^,^14^,^15^,^16^,^17^,^18^,^19^,^20^,^21^,^22^,^23^,^24^,^25^,^26^,^27^,^28^,^29^,^30^,^31^,^32^,^33^,^34^,^35^,^36^,^37^,^38^,^39^,^40^,^41^, in which different subsets of the completely identical oscillators exhibit completely distinct dynamical behaviors, e.g., synchronization or incoherent oscillations. In the past decade, chimera states were observed in, e.g., regular networks of phase-coupled oscillators with ring topology^7^,^8^,^9^, regular networks hosting a few populations^10^,^15^, and two-dimensional lattice^6^,^16^ or torus^34^,^21^. Issues that were addressed include transient behavior of chimera states^17^,^18^,^19^, control^26^, the effects of time delay^14^,^11^,^40^, phase lags^22^, and coupling functions^27^,^28^,^29^. Theoretically, two approaches were developed to analyze and understand the dynamical origin of chimera states: self-consistency equation^7^,^8^,^9^ and partial differential equation (PDE)^42^,^43^. Quite recently, the effects of random perturbation and complex topologies of coupling on chimera states were investigated^23^,^30^,^37^. Experimentally, chimera states were observed in a system of chemical oscillators^24^,^31^, in an optical system^25^, in coupled mechanical oscillators^32^, and in electrochemical systems^33^,^36^. Other natural phenomena such as unihemispheric sleep^44^,^45^, neural spikes^46^,^47^, and ventricular fibrillations^48^ are among those associated with chimera states. We note that, while the term of chimera states first appeared about a decade ago^7^,^8^, their signatures were actually observed earlier^49^ from the spatiotemporal evolution of a system of coupled nonlinear oscillators and the phenomenon was named “domain-like spatial structure”.

While a chimera state is commonly referred to as the situation where two dynamically distinct states coexist in different regions of the physical space, in certain particular settings more than two coexisting states can occur, e.g., in systems with time delay^11^,^14^, phase lags^22^, or special coupling functions^27^,^28^,^29^. Such a situation is typically characterized by the emergence of multiple clusters in the physical space, each being associated with a specific region. For convenience, we use the name “multicluster chimera states”. Because of the special system setting required for such states to occur, their generality or “typicality” in realistic physical systems becomes an interesting issue.

In this paper, we demonstrate that multicluster chimera states can occur commonly in the “traditional” setting of Kuramoto networks of phase coupled oscillators, without the need to impose special dynamical features on time delay, phase lag, or special coupling function differing from that associated with the classical Kuramoto model. In fact, perturbing the coupling configuration^23^,^30^ can lead to the emergence of various multicluster chimera states with rich spatiotemporal dynamical patterns. In particular, starting from the classical, non-locally coupled Kuramoto oscillator network, we systematically remove a small number of links. As the fraction of the removed links is increased from zero, chimera states with different number (denoted by *m*) of clusters emerge, i.e., become stable, and then disappear (become unstable). An interesting phenomenon is that, certain *m*-cluster chimera states can undergo a period-doubling like bifurcation to states with 2*m* clusters. We propose a phenomenological theory, based on the intuitive idea of mutual enhancement among oscillator subsets exhibiting similar dynamical behaviors in space, to explain the “bifurcation” behavior of chimera states with distinct spatiotemporal patterns. The theory predicts correctly key features such as the emergent order of *m*-cluster chimera states, the corresponding region of the topology parameter, and the possible *m* values for the occurrence of cluster doubling. Our results imply that multicluster chimera states can occur in non-locally coupled oscillator networks more commonly than previously thought.

## Results

### Model

**Figure 1:**
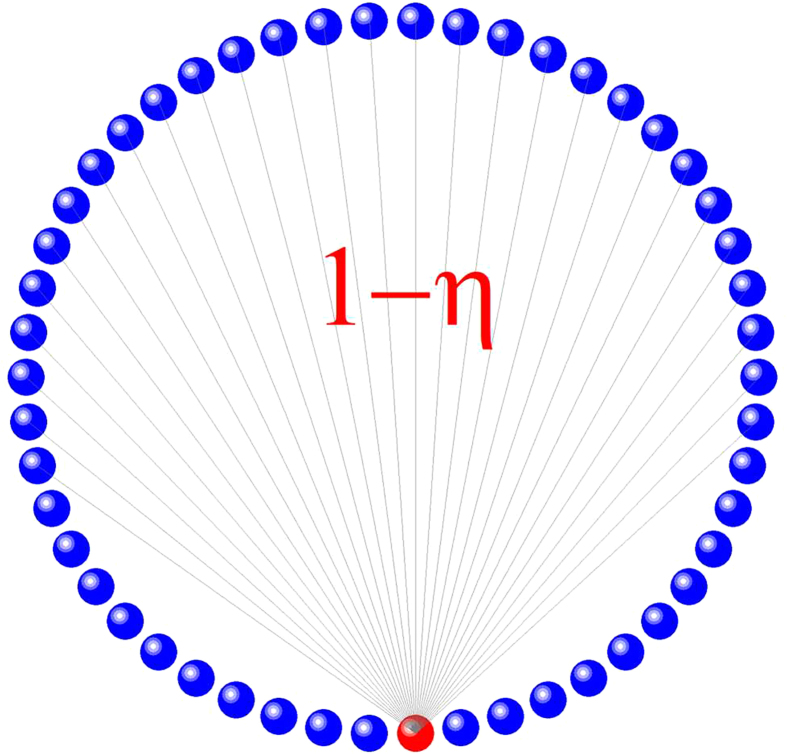
Nodal connection structure after link removal. For any node in the network, removal of links starts from the nodes with the minimum distance to it (colored by red).

We consider a one-dimensional network of *N* non-locally coupled, identical phase oscillators with periodic boundary condition (the ring configuration). The system is mathematically described as


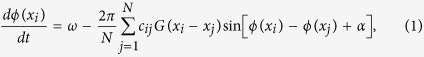


where *ϕ*(*x*_*i*_) is the phase of the *i*th oscillator at position *x*_*i*_. For convenience, we choose the range of the spatial variable to be [−*π*,*π*]. Since the oscillators are identical, the natural velocity and phase lag parameter, *ω* and *α*, respectively, are chosen to be constants that do not depend on the spatial location of the oscillator. Without loss of generality, we set *ω* = 0 and choose *α*  <  *π*/2. The kernel *G*(*x*_*i*_ − *x*_*j*_) = [1 + *A*cos(*x*_*i*_ − *x*_*j*_)]/(2*π*) is a non-negative even function that characterizes the non-local coupling among all the oscillators. The quantity *c*_*ij*_ is the *ij* th element of the *N* × *N* coupling matrix *C*, where C_*ij*_ = 1 if there is coupling from the *j*th oscillator to the *i* th oscillator, and C_*ij*_ = 0 indicates the absence of such coupling. We systematically remove certain fraction of links from every node, while ensuring that all nodes remain identical and structurally indistinguishable. To do this we introduce a tunable topological parameter *η* = 2*L*/*N* (*L* = 1, . . . , *N*/2), the fraction of neighbors removed for any given oscillator, where *L* denotes the number of removed links from each side of the node. We have C_*ij*_ = 0 for *j* = *i* − *L*, . . . , *i* + *L*. The connection pattern of a node after link removal is shown in Fig. 1, where the node was originally connected to all other nodes in the network, and link removal is carried out in the order of increasing distance from this node.

The network dynamics can be characterized by the following complex order parameter *Z*, defined^7^ for oscillator *i* as


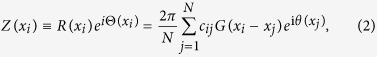


where the phase of the oscillator is written as *θ* = *ϕ* − Ω*t*, and Ω denotes the velocity of the oscillators in the coherent subset when a chimera state emerges. Theoretical insights into the chimera states can be obtained by resorting to the continuum limit *N* → ∞ to reduce the system to one described by PDE^42^,^43^, where the state of the system is characterized by a probability density function *f*(*x*, *ϕ, t*) that satisfies the continuity equation


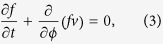


and *v* is phase velocity^13^. The function *f*(*x*, *ϕ, t*) can be expressed in terms of Fourier series expansion as


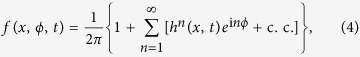


where “*c*.*c*.” stands for the complex conjugate of the previous term, and the *n*th coefficient is the *n*th power of some function *h*(*x*, *t*) that effectively characterizes the state of the system. The time evolution of *h*(*x*, *t*) associated with the order parameter *Z*(*x*, *t*) is^42^,^43^


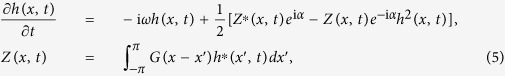


where *G*(*x* − *x*′) is a piecewise-smooth coupling function


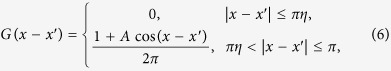


taking into account the deletion of the nearby *η* fraction of couplings. The modulus of the order parameter, 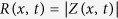, can be obtained from the numerical solution of Eq. (5).

### Numerical findings and interpretation

**Figure 2:**
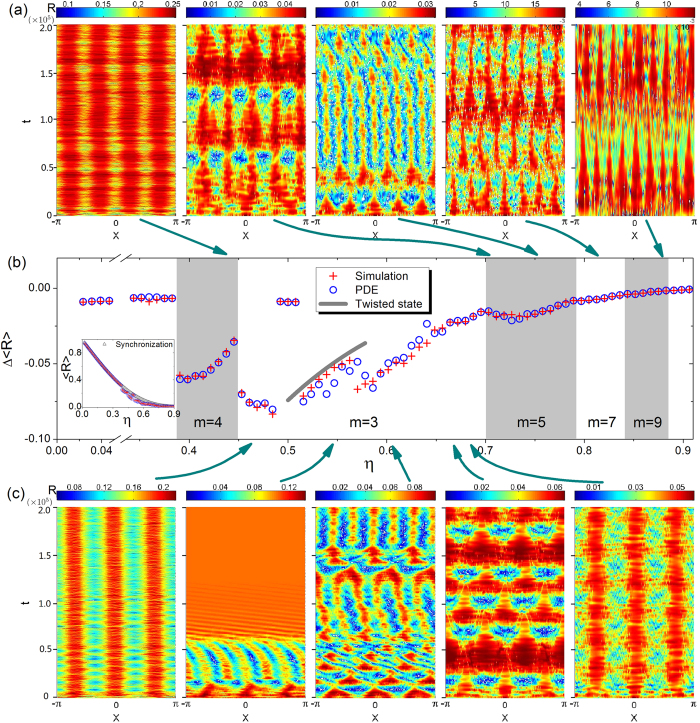
Spatiotemporal patterns associated with multicluster chimera states after link removal. (**a**,**c**) Typical spatiotemporal patterns of the order parameter *R* for different values of *η*, a parameter characterizing the extent of link removal. The magnitudes for different panels are indicated using the respective color bars. The results are obtained for parameters *A* = 0.995 and *α* = 1.39 and system size *N* = 256, and for over 2 × 10^5^ time steps. The values of *η* for different panels are (**a**) 0.43, 0.70, 0.75, 0.80, 0.87, and (**c**) 0.46, 0.54, 0.59, 0.66, 0.68 from left to right, respectively. (**b**) Difference in the order parameter, 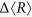, between the *m*-cluster chimera state and the corresponding hypothetical synchronous state, where the values of the average order parameter 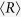 are displayed in the inset. The order parameters calculated through direct simulation of Eq. (1) (red pluses) and from the PDE Eq. (5) (blue circles) agree well with each other. The number of clusters emerged as *η* is increased is *m* = 4, 3, 5, 7, 9, and so on, and they are distinguished using white and gray backgrounds.

Numerically, we observe a variety of rich phenomena when links are systematically removed. In particular, using the real order parameter *R*(*x, t*), we can identify the emergence of multiple cluster chimera states, where each cluster corresponds to a coherent group of oscillators. Figure 2 shows the spatiotemporal patterns of the emergent *m*-cluster chimera states for different intervals of *η*, which indicates that the emergence of the chimera-state patterns is robust with respect to reasonable variations of these parameters. In the simulations, the system parameters are *A* = 0.995 and *α* = 1.39, and the initial condition is generated^8^,^9^ using the function *ϕ*(*x*) = 6*r* exp(−0.76*x*^2^), where *r* is a random variable uniformly distributed in [−1/2,1/2]. In fact, the results obtained from direct simulations of Eq. (1) for finite-size networks and from the PDE approach [Eq. (5)] in the continuum limit *N* → ∞ agree with each other with similar spatiotemporal patterns. As shown in the inset of Fig. 2(b), the degree of synchrony as characterized by 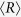 for different *η* values differs by orders of magnitude. For clarity, we use different color bars to distinguish the magnitudes of the spatiotemporal patterns in different panels. As *η* is increased, the number *m* of clusters undergoes changes from 4 to 3 (or 3&6), to 5 (or 5&10), to 7, and to 9, etc. Here the 3&6 state (or the 5&10 state) is a state that switches between 3-cluster and 6-cluster (or between 5-cluster and 10-cluster) chimera behaviors.

To better understand the impact of multicluster chimera states on global coherence of the system, we calculate the average order parameter 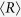 over time and space. For a hypothetical system of the same structure but exhibiting global synchronization, 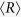 is given by


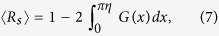


which serves as a reference to characterize the system’s coherence. We can then examine the difference 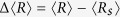, in different regions of *m* [differentiated using different background colors in Fig. 2(b)], to quantify the degree of coherence as compared with the synchronized reference state. In general, the coherence of the *m*-cluster chimera state is weaker than that of the global synchronization state, so the maximum value of 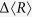 is zero.

**Figure 3:**
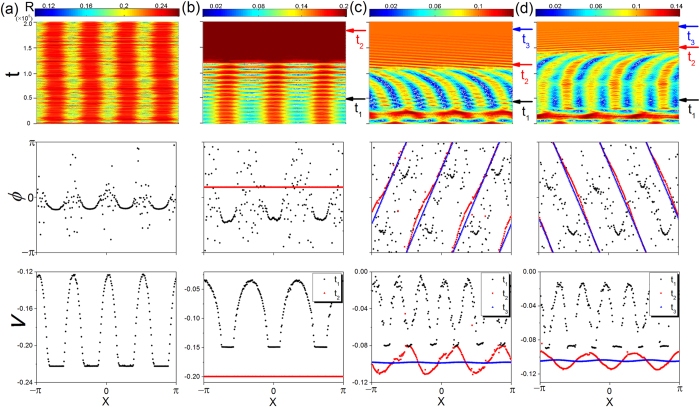
Different final states associated with a multicluster chimera state. For the ring network system of size *N* = 256, spatiotemporal patterns of the order parameter *R*, instantaneous phase *ϕ* and short-term average velocity *v* for: (**a**) a stable 4-cluster chimera state, (**b**) a transient 3-cluster chimera state evolving into a globally synchronous state, and (**c**,**d**) transient 3-cluster and 6-cluster chimera states evolving into a 6*π*-twisted state, respectively. The phase *ϕ* at several instants and the corresponding short-term average phase velocity *v* (temporal average over a time window with length 10^4^) are displayed to demonstrate the dynamical processes, e.g., global synchronization at *t*_2_ in (**b**), 6-cluster chimera states at *t*_1_, and the twisted states at *t*_3_ in (**c**,**d**). The results in (**a**–**d**) are obtained from Eq. (1) for *η* = 0.43, 0.48, 0.55, and 0.54, respectively.

In the small neighborhood of zero *η* value, the observed states are conventional chimera states consisting of a coherent and an incoherent clusters. For *η* ~ 0.4, 4-cluster chimera states emerge. In the 4-cluster region [*m* = 4 region in Fig. 2(b)], the value of 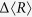 increases with *η*, which can be attributed to the increasing fraction of coherent groups, as demonstrated by the red color in the spatiotemporal patterns [first panel in Fig. 2(a)]. The behaviors in subsequent parameter regions are richer and more complicated. In particular, the 3-cluster chimera states for small values of *η* are stable and regular as the 4-cluster chimera states. As *η* is increased further, the 3-cluster configuration becomes unstable and evolves eventually to global synchronization. In the 3-cluster region, various other states can emerge, which include (in successive order) stable regular 3-cluster states, transient 3-cluster states toward global synchronization, 6*π*-twisted states and 3&6 cluster double-state switching process, cluster drift states, and so on. One remarkable phenomenon is spatial period doubling (or spatial cluster doubling) in the 3-cluster region, in which each cluster bifurcates into two clusters and a 6-cluster chimera state emerges consequently, as shown in Fig. 2(c) (2nd and 3rd panels). The 6-cluster chimera states are unstable and can evolve into 6*π*-twisted states, as shown in the 2nd panel in Fig. 2(c), which will be further discussed in Fig. 3. Analogous to chemical oscillating reactions^50^, self-organized double-state switching processes are observed, in which the 3-cluster and 6-cluster chimera states appear and disappear alternatively, leading to spatiotemporal patterns of switching between the two states. The switching process also takes place in the 5-cluster region, where the system alternates between 5-cluster and 10-cluster chimera states, as shown in the 3rd panel in Fig. 2(a). Overall, as *η* is increased in the 3-cluster region, the resulting state is a *cluster drifting state* with strong intrinsic correlation in the spatiotemporal dynamics, as characterized by harmonically temporal breathing and spatial drifting of the coherent and incoherent groups [4th panel in Fig. 2(c)].

Chimera states with *m* = 5, 7, and 9 clusters emerge as *η* is increased further. In the 5-cluster region, the breathing and spatial period doubling phenomena are also present, as shown in Fig. 2(a) (2nd and 3rd panels). At the boundary between the two neighboring *m* regions shown in Fig. 2(b), the system with different initial phase configurations can evolve into either of the two *m* states, leading to fuzziness of the boundary.

As shown in Fig. 2, the destinations of the system can be a stable chimera state, or transient and finally reaching a globally coherent state such as synchronization or a coherent twisted state. Deviations from the structures that sustain the *m*-cluster patterns will make the *m*-cluster chimera state transient (a detailed analysis will be given in **Methods**). Figure 3 shows the spatiotemporal patterns of the order parameter *R*, instant phase *ϕ* and the average velocity *v* associated with a stable chimera state, a transient 3-cluster chimera state evolving into global synchronization, and two examples of transient 3&6-cluster chimera states evolving into coherent 6*π*-twisted states^51^ that are phase-locked states with the phase difference between neighboring oscillators on the ring to be 2*mπ*/*N*. From the patterns of the order parameter *R*(*x*, *t*) in Fig. 3(b–d), we see that the oscillators in the globally coherent state have a identical constant value of *R*. The *R* values associated with the twisted states are smaller than that associated with global synchronization. The order parameter of an ideal twisted state can also be obtained from Eq. (2), and the difference 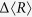 from that of a synchronous state is plotted in Fig. 2(b) (gray solid curve) in the *m* = 3 region.

The heuristic reason that a transient chimera state can evolve into either a globally synchronous state or a coherent phase-twisted state can be seen, as follows. The coherent groups (separated by the incoherent groups) in the *m*-cluster chimera state (with *m* = 4, 3, 5, 7 and so on) are found to be synchronized with each other. However, for the 2*m*-cluster chimera state “bifurcated” from the *m*-cluster chimera state, each pair of the nearby coherent groups have opposite phase *ϕ* but the same velocity *v*. Intuitively, for the first case of *m* synchronized clusters, global coherence of the system tends to increase when the coherence groups are enlarged, and the incoherent oscillators will consequently join the synchronized groups. As a result, global synchronization finally sets in, replacing the *m*-cluster chimera state. For the case of coherent phase-twisted state, the 2*m*-cluster chimera state is composed of opposite-phase coherence groups with large phase differences, as exemplified in Fig. 3(c,d) at time *t*_1_. The interaction between the coherent and incoherent groups can cause the phases of the oscillators to have uniform and ordered arrangement in each of the *m* clusters so that the 2*mπ*-twisted state will finally replace the 2*m*-cluster chimera state.

## Discussion

The discovery of the counterintuitive phenomenon of chimera states in coupled dynamical networks was remarkable^6^,^7^,^8^,^49^. In a spatially extended system of coupled, completely identical oscillators, depending on the coupling parameter the oscillators form two distinguished groups in *space*, where one group exhibits a highly coherent behavior while oscillators belonging to the complementary group are incoherent. The coherent and incoherent behaviors emerge as a *single* state of the underlying dynamical system, which is quite different from the phenomenon of multistability in nonlinear dynamical systems^52^,^53^. Often, a nonlinear dynamical system can exhibit multiple coexisting attractors, each with its own basin of attraction. Starting from a random initial condition the system approaches one particular attractor that can be a stable fixed point, periodic, quasiperiodic, or chaotic. The key difference from the chimera states is that, from a single initial condition the asymptotic state of the system cannot simultaneously exhibit more than one of these traits. Most existing works on chimera states focused on the setting of fully connected, non-local coupling configurations, in which the oscillators of the system typically are self-organized into a coherent and an incoherent groups. The question that we address in this paper is what can happen to the chimera states when structural deviations from the fully connected coupling configuration occur in a systematic fashion.

Our main finding is that, as links are removed from the network in an orderly fashion, multicluster chimera states can emerge. Especially, for any node in the network, we systematically remove a given fraction of links, starting from the nearest neighbors. The network is still regular under such structural changes, because the number of links remains identical for every node. A surprising result is that, as the fraction of the orderly removed links is increased, chimera states consisting of different numbers of spatial clusters are observed in different intervals of the link-removal parameter. While the order of emergence of such distinct chimera states appears to be somewhat irregular, we find that it can be explained by the mechanism of enhanced or reduced interactions among different groups of oscillators through a phenomenological theory (see **Methods**). Especially, by hypothesizing a simple, binary type of interaction between any pair of oscillators, we can determine the number of clusters embedded in the chimera state for any given value of the link-removal parameter, with remarkable agreement with the numerical results.

We note that, when links are randomly removed from the network so that it becomes somewhat random, in a statistical sense chimera states can persist if the fraction of the removed links is relatively small^23^,^30^. In such a case the observed chimera states consist typically of two clusters. In this regard, a recent work by Omelchenko *et al.*^54^ studied the robustness of chimera states for coupled FitzHugh-Nagumo oscillator networks. The main finding is that gaps in the coupling matrix can result in a change in the multiplicity of the incoherent regions associated with the chimera state. However, to our knowledge, the *orderly* emergence of multicluster chimera states under systematic link removal, as uncovered in this paper, has not been reported before. It would be interesting if such exotic chimera states can be observed in experiments.

## Methods

We develop a phenomenological theory to understand the emergence of multicluster chimera states and their stabilities. As we find numerically, the clusters emerge according to the order *m* = 4, 3, 5, and a few subsequent odd numbers as the parameter *η* is increased. Through extensive simulation with different initial phase configurations, we observe that *mutual enhancement* between coherent (or incoherent) groups of oscillators in the network is key to emergence of multicluster chimera states.

**Figure 4:**
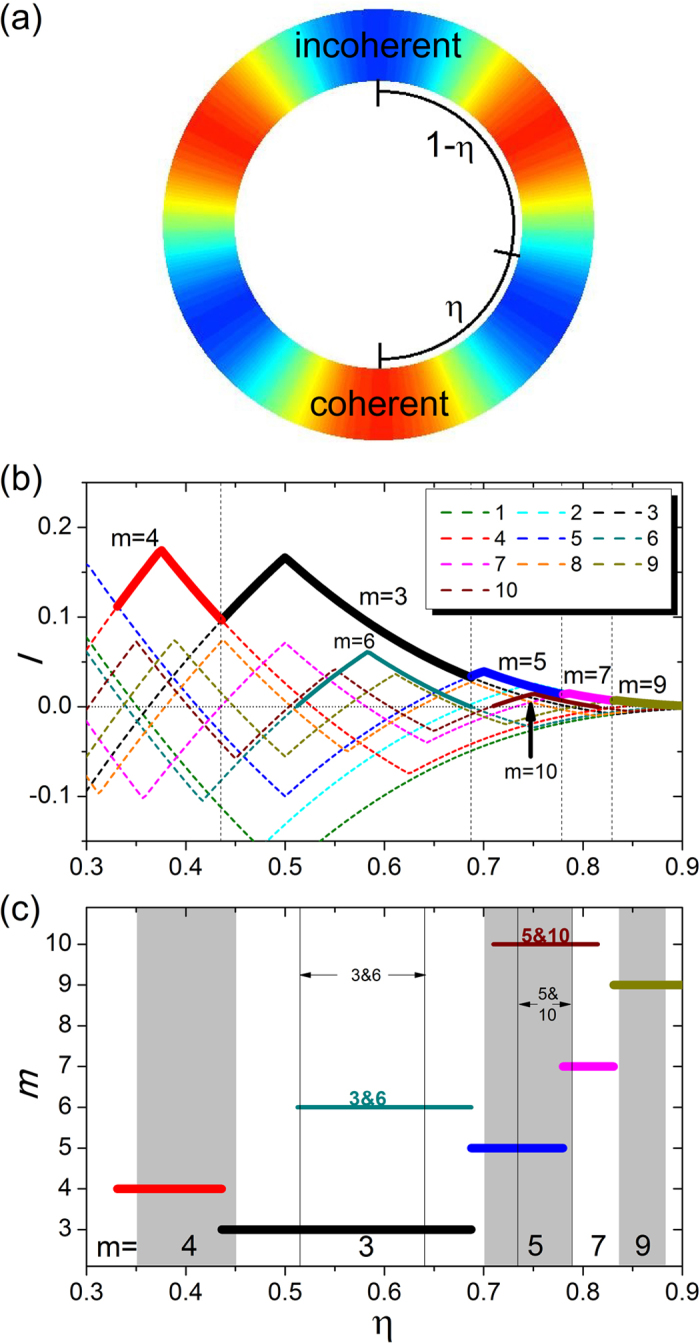
Mutual enhancement and formation of multicluster chimera states. (**a**) Schematic illustration of *m* = 3 cluster chimera states in certain range of *η*. The colors represent the values of the order parameter *R* and the regions centered with red and blue colors correspond to coherent and incoherent groups of oscillators, respectively. The reference oscillator for calculating the enhancement factor *I* is the bottom node at the center of the coherent group. (**b**) Enhancement factor *I* for the reference oscillator as a function of *η*, for different values of *m*. The regions of maximum *I* values among the different *m* curves are specified as bold lines, which can be regarded, approximately, as the regions in which the corresponding *m*-cluster chimera states emerge. (**c**) Regions of *m*-cluster chimera states predicted by our mutual-enhancement theory (the colored thick horizontal lines at different *m* levels), including the main regions *m* = 4, 3, 5, 7, and 9, and the two subregions with 3&6-cluster and 5&10-cluster chimera states. The white and gray backgrounds have the same meaning as those in Fig. 2, i.e., they denote the regions of *m*-cluster chimera states, which are obtained from both simulation of Eq. (1) and solution of Eq. (5). The subregions for 3&6-cluster and 5&10-cluster chimera states obtained from Eqs (1) and (5) are also specified with the thin black vertical lines and the corresponding notations.

To gain insight into the mechanism of mutual enhancement, we analyze the stability of the coherent (or incoherent) groups in an idealized *m*-cluster chimera state. For a given *coherent* group, the contribution to the coupling from oscillators in other *coherent* groups tends to stabilize the state (a positive effect), while that from oscillators in the *incoherent* groups plays the opposite role (a negative effect). For an incoherent group, the effects of other coexisting coherent and incoherent groups are negative and positive, respectively. That is, oscillators in the *like* groups (coherent versus coherent or incoherent versus incoherent) tend to enhance each other’s stability, while those in the *unlike* groups (coherent versus incoherent or vice versa) tend to destabilize each other. To be concrete and quantitative, we define an enhancement factor *I*(*η*) that depends on the system parameter *η* and assume that, for an oscillator in the coherent group, the contribution from each coherent-group oscillator is +1, while that from an incoherent-group oscillator is −1. Consider the oscillator at the center of a coherent group, e.g., the bottom oscillator in Fig. 4(a). The total contribution from other oscillators to the enhancement factor for this oscillator is


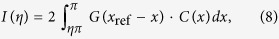


where the contribution of the oscillator located at *x* is *C*(*x*) = ±1, depending on whether the oscillator at *x* is in a like or an unlike group with respect to the group of the reference oscillator located at *x*_ref_, and the coupling kernel *G*(*x*_ref_ − *x*) is effectively the weight of the contribution. Figure 4(b) shows the enhancement factor *I* associated with the reference oscillator as a function of *η*, calculated from the patterns of different *m*-cluster chimera states. In addition, the enhancement factor *I* of the oscillator at the center of an *incoherent* group exhibits the same behavior. The mutual enhancement factor is increased (or decreased) as more (or fewer) groups of the same kind are involved for different values of *η*.

The dependence of the maximum enhancement factor, *I*_max_, among those for different *m* values on the parameter *η* are marked by the bold curves in Fig. 4(b). We see that the variation of *I*_max_ follows the same sequence as that for emergence of *m*-cluster chimera states, i.e., *m* = 4, 3, 5, 7, and so on. For a given coupling structure as determined by *η*, the pattern with the maximum enhancement factor will “stand out” in the competition among patterns of different *m* values. The estimated *η* region for each *m*-cluster chimera state can be predicted through the corresponding region of each *m* with the maximum enhancement factor. Figure 4(c) shows the estimated regions for each *m*-cluster chimera state (thick straight lines) and the corresponding regions obtained through direct simulations and PDE (gray and white backgrounds as in Fig. 2). We see that our estimation of the parameter region in which *m*-cluster chimera states occur based on the maximum enhancement factor agrees well with the simulation results, including the order of *m* that emerges with increased *η* and the region of *η* for each *m*. The agreement indicates that our phenomenological theory based on mutual enhancement to explain the occurrence of *m*-cluster chimera state captures the essential dynamics of the emergence of the exotic states.

Our theory is also effective at predicting the emergence of 2*m*-cluster chimera states from the *m*-cluster background, through the behavior of the second-largest enhancement factor, where the corresponding state can emerge when it possesses the spatial symmetry as that of the maximum-*I* state. For example, for the *m* = 3 maximum-*I* region, the second-largest *I* cluster is associated with the *m* = 4, 7, 10, 6, and 5 states as *η* is increased. However, only the *m* = 6 state has the same spatial symmetry as that of the *m* = 3 background state. For the *m* = 5 maximum-*I* region, the second-largest *I* states are *m* = 3, 10, and 7, but the state that has the same spatial symmetry as that of the *m* = 5 state is the *m* = 10 state. As shown in Fig. 2, the emergence of 6-cluster (or 10-cluster) chimera states from the 3-cluster (or 5-cluster) background chimera states is observed. However, for the *m* = 4 region, the second largest values of *I* correspond to the *m* = 5, 9, and 3 states that have different spatial symmetry than that of the *m* = 8 state. As a result, no 2*m*-cluster can arise from the *m* = 4 state.

The fuzzy boundary between different *m* regions in Fig. 2 can be understood based on our mutual enhancement theory: at the boundary the *m*-cluster chimera state no longer possesses the maximum *I* value. For example, around the boundary of *m* = 4 and 3, the two chimera states have similar values of *I*, and therefore both are likely to emerge.

Since the coupling structure of the oscillator system, as controlled by the parameter *η*, is regular, theoretical analysis of pattern formation can be carried out by using the continuity equation Eq. (3) and the concept of invariant manifold^42^,^43^. In this approach, various multi-cluster patterns correspond to rotating wave solutions of the underlying infinite-dimensional dynamical equation. A systematic analysis^39^ led to a number of results with respect to the general coupling function *G*(*x*) in Eq. (1). For example, every non-zero harmonic term in the Fourier series of *G*(*x*) gives rise to a number of solutions. A more recent work^41^ discussed the simple case where *G*(*x*) is a purely harmonic function, e.g., cos(*kx*), or a superposition of two harmonics in the form of cos(*kx*) + cos[(*k* + 1)*x*], with *k* being an arbitrary positive integer. The piecewise smooth coupling function *G*(*x*) employed in our present work, however, consists of an infinite number of harmonics. In this case, it is not clear whether a mathematical theory can be developed to analyze the pattern formation process. Because of this difficulty, we resort to developing a phenomenological theory, in which the value of *I*(*η*) effectively determines, in a self-consistent manner, the emergence of *m*-cluster patterns in the continuum limit, as exemplified in Fig. 4. This approach yields results that agree well with those from direct numerical simulations, despite the fact that our analysis based on *I*(*η*) takes into account only the coarse-grained configuration of multi-cluster patterns formed due to the mutual interactions between oscillators in distinct spatial regions.

**Figure 5:**
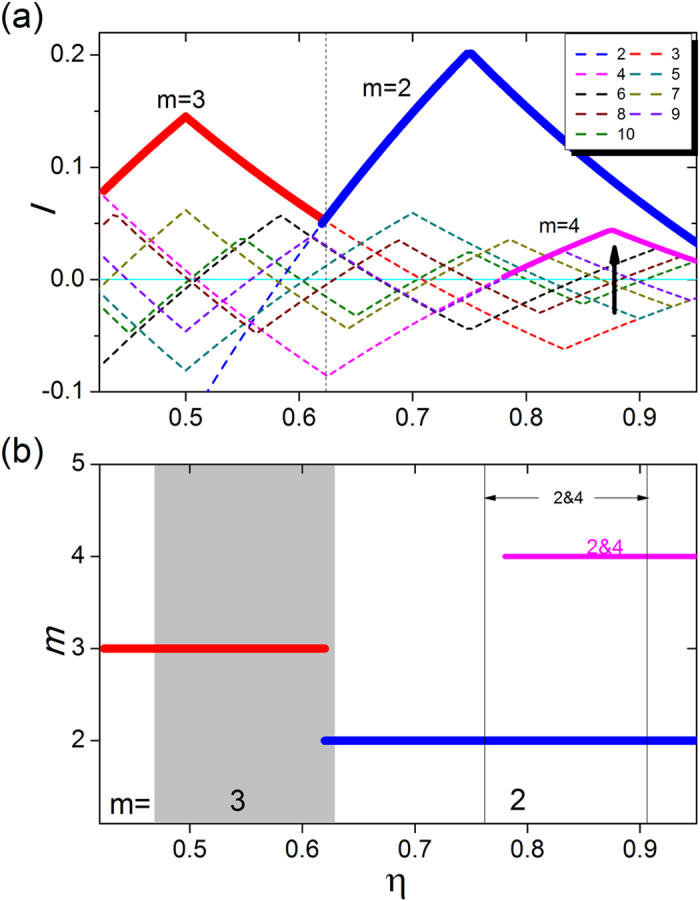
Enhancement factor and predicted regions of multicluster chimera states for exponential coupling kernel. (**a**) Enhancement factor *I* as a function of *η* for different values of *m*. The regions of maximum *I* values among the different *m* curves (specified as bold lines) are the regions in which the corresponding *m*-cluster chimera states emerge. (**b**) Regions of *m*-cluster chimera states predicted by the mutual-enhancement theory (the colored thick horizontal lines at different *m* levels), including the main regions *m* = 3 and 2, and the subregion 2&4 for the 2&4-cluster chimera states. The gray and white backgrounds denote the regions of the *m*-cluster chimera states obtained from Eq. (1) and Eq. (5), respectively. The subregion for the 2&4-cluster chimera states obtained from Eqs (1) and (5) is within *η* = 0.76 and 0.91 as marked by the two thin black vertical lines. The exponential coupling kernel has *κ* = 4, and the phase lag is *α* = 1.457.

**Figure 6:**
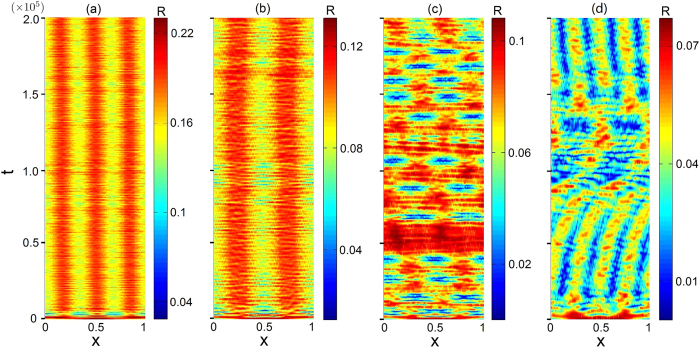
Representative spatiotemporal patterns for exponential coupling kernel. The values of *η* for (**a**–**d**) are 0.51, 0.67, 0.72, and 0.79, respectively. The exponential coupling kernel has *κ* = 4, and the phase lag is *α* = 1.457.

To demonstrate the general applicability of our mutual-enhancement theory with respect to the choice of different coupling kernels, we have studied the case of normalized exponential coupling kernel


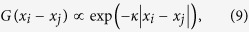


where *x*_*i*_ and *x*_*j*_ run from 0 to 1 with periodic boundary condition^7^. Figure 5(a) presents the curves of *I*(*η*) from Eq. (8) with integral interval [*η*, 1], while Fig. 5(b) compares the prediction (the colored thick horizontal lines) with results from simulations of Eq. (1) (the gray and white regions). We observe a good agreement. The corresponding spatiotemporal patterns for a representative set of *η* values (0.51, 0.67, 0.72, and 0.79) are shown in Fig. 6.

**Figure 7:**
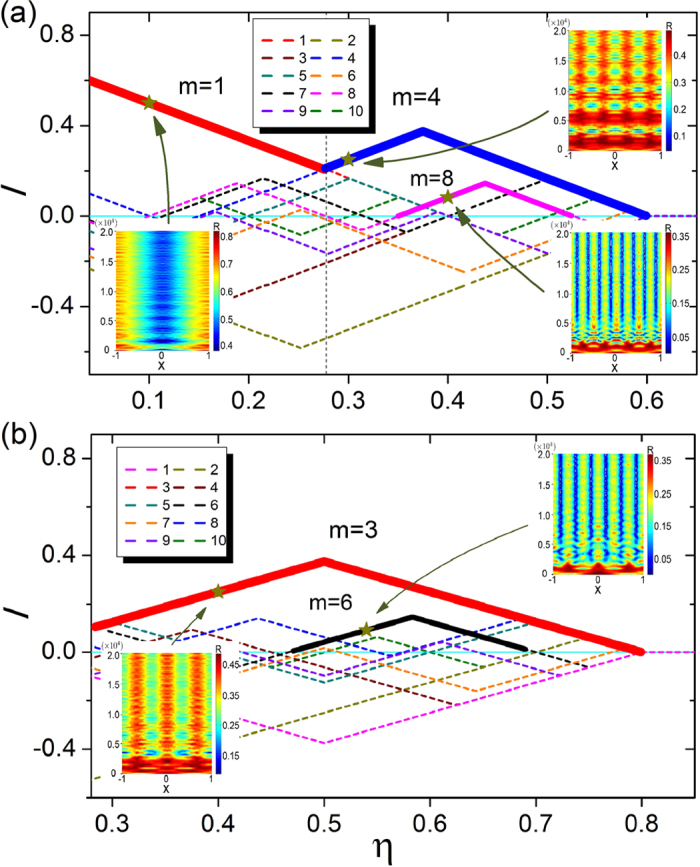
Enhancement factor and predicted regions of multicluster chimera states for rectangular coupling kernel. Enhancement factor *I* as a function of *η* for different values of *m*. The regions of maximum *I* values among the different *m* curves (specified as bold lines) are the regions in which the corresponding *m*-cluster chimera states emerge. The results in (**a**) are for *N* = 1024 and *γ* = 0.6, and those in (**b**) are for *N* = 512 and *γ* = 0.8.

Furthermore, we have studied the case of rectangular coupling kernel


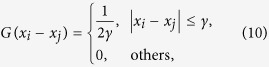


where *x*_*i*_ and *x*_*j*_ run from −1 to 1 with periodic boundary condition, and 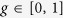 is a parameter characterizing the width of the coupling range for oscillators. The behaviors of *I*(*η*) and a number of typical spatiotemporal patterns are shown in Fig. 7(a,b), for *γ* = 0.6 and 0.8, respectively. The results are essentially the same as those for the case of sinusoidal coupling function, demonstrating the general applicability of our mutual-enhancement theory.

## Additional Information

**How to cite this article**: Yao, N. *et al.* Emergence of multicluster chimera states. *Sci. Rep.* **5**, 12988; doi: 10.1038/srep12988 (2015).
